# Supplementary material for: Are carbon emissions trading and green financial instruments synergistic? -Comprehensive quantitative research based on content analysis
Source: PLoS One. 2024 Mar 7;19(3):e0298601. doi: 10.1371/journal.pone.0298601 (PMC10919601; doi:10.1371/journal.pone.0298601)
Supplement: S1 Data — (ZIP) [file pone.0298601.s001.zip › Data/地级市Z_F工作报告.zip!/╕╩╦α/╒┼╥┤╩╨/└┤╫╘╣1⁄2╓┌║┼í╛┬φ┐╦╩2╛▌═°í┐_2016.docx]

1各位代表：

　　现在，我代表张掖市人民政府向大会报告工作，请予审议，并请各位政协委员和列席人员提出意见。

　　关于“十二五”和2015年工作

　　过去的五年，是全力实施“十二五”规划、推动经济社会转型跨越发展、加快全面建成小康社会进程不平凡的五年。五年来，面对错综复杂的国内外环境和艰巨繁重的改革发展任务，我们在省委、省政府和市委的坚强领导下，坚持以科学发展观统揽全局，认真贯彻落实党的十八大及十八届三中、四中、五中全会和习近平总书记系列重要讲话精神，坚持协调推进“四个全面”战略布局，坚持把发展生态经济作为转变发展方式的基本途径、把建设生态经济功能区作为经济结构战略性调整的主攻方向，紧紧围绕“四二”部署、实施“六六”战略，持续强化基础建设、生态建设、文化建设和产业培育，积极适应经济发展新常态，有效应对各种风险和挑战，经济平稳快速增长，综合实力持续增强；优势主导产业增量扩容，结构持续优化；生态建设全面加强，比较优势持续扩大；基础设施明显改善，城乡面貌发生深刻变化；精准扶贫成效显著，民生保障改善更加有力；开放开发步伐加快，社会文明和谐程度不断提高。“十二五”规划主要目标任务顺利完成。

　　（一）着力强化项目带动，经济社会发展动力持续增强。坚持把发展作为第一要务，把项目作为第一支撑要素，把招商引资作为项目建设的持久动力和源头活水，实施各类项目2600个，其中亿元以上项目400个，累计完成固定资产投资1140亿元，比“十一五”增长1.6倍，年均增长20%。招引项目962个，到位资金先后跨过100亿、200亿、300亿、400亿四个台阶，累计达到1197亿元。金融机构存贷款余额达到564亿元、486亿元，较“十一五”末分别增长114%和268%，存贷比从2010年的50%提高到86%。主要经济指标增速高于全省、全国平均水平，生产总值年均增长10.5%，大小口径财政收入实现翻番，人均GDP突破3万元。全面建成小康社会进程加快，实现程度达到76.7%，比“十一五”末提高11.6个百分点，居全省第5位，率先实现全面小康的优势明显。全国小微企业创业创新基地城市示范、张掖国家级经济技术开发区、绿洲现代农业示范区、公共文化服务示范区、生态安全屏障综合试验区等一系列国家级示范项目成功获批，加快发展的基础更为坚实。

　　（二）着力强化转型升级，主导产业规模效益大幅提升。坚持把转方式、调结构作为推动跨越发展的重中之重，大力发展特色优势产业，推动三次产业转型升级、协调发展。坚持绿色、循环、效益、品牌发展理念，加快建设现代农业大市。种植业、养殖业、菌业三元双向循环模式基本形成。农业投资年均增长27.4%，粮食生产实现十四连增。农业增加值年均增长5.7%，高于全国、全省平均水平。国家玉米制种基地建设项目获批实施，建成国家级玉米种子产业园和电子交易平台。“张掖玉米种子”、“张掖肉牛”、“张掖葡萄”等获全国地理标志证明商标和农产品地理标志登记保护，绿色有机农产品、标准化生产和农业机械化水平稳步提高。现代畜牧业扩容增量，规模化养殖优势凸显，畜产品加工实现重大突破，沅博、博峰、前进牧业等116户骨干企业建成投产。流转土地116.7万亩，农民专业合作社、家庭农场、家庭林场分别达到5554家、1500家和242家。农产品加工转化率提高到60%。坚持走资源循环、企业聚集的生态工业发展之路。着力改善工业发展的基础条件，张掖工业园区晋升为国家级经济技术开发区，民乐生态工业园区基础设施水平明显提升，2+4园区体系基本形成，园区对工业的综合承载能力显著增强。完成工业固定资产投资408亿元，年均增长23%；工业增加值年均增长12.8%；规上企业由117户发展到210户，实现增加值63亿元，年均增长12.4%。“两新一特”产业体系初步建立，形成10万千瓦风电、80万千瓦光电、100万千瓦水电装机容量和60万吨钨选、80万吨锰铁、60万吨铜选、500万吨煤炭、300万吨农产品加工生产能力。15个产品获甘肃名牌产品称号，滨河集团获省政府质量奖。整合关闭煤矿64户，淘汰落后产能230万吨，万元生产总值能耗年均下降3.65%。坚持旅游与文化体育深度融合发展战略，加快发展旅游文化为龙头的现代服务业。游客接待人数、综合收入达到1505万人次和76亿元，较“十一五”末分别增长8倍和8.1倍，年均增长34%和38%，旅游综合收入占GDP比重由“十一五”末的4.3%提高到20%。市县两级落实旅游发展专项资金6亿元，拉动投资130亿元，丹霞国家地质公园、景观大道等188个重点项目建成运营。成功举办“湿地之夏·金张掖”旅游文化艺术节、祁连山国际超百公里山地户外运动挑战赛、中国汽车拉力锦标赛等品牌赛事和重大节会。张掖被确定为全国体育产业联系点。祁连玉文化产业园命名为国家级文化产业示范基地，文化产业增加值占GDP的比重由“十一五”末的1%提高到2.2%。现代物流、健康养老、电子商务等新兴业态发展加速，三次产业比重由“十一五”末的29.3∶35.5∶35.2调整到25.5∶30∶44.5，经济结构持续优化。

　　（三）着力强化基础设施，城乡面貌发生深刻变化。坚持把夯基础、促发展、树形象作为城乡建设的重要目标，全力加快城乡一体化进程。两个1+5城市框架体系基本形成，城市建成区面积达到95.9平方公里，较“十一五”末翻了近一番，城镇化率达到42.19%，较“十一五”末提高7.4个百分点。滨河新区及五县新城区建设高起点、高标准推进，旅游城市功能定位更加清晰。改扩建城区道路155.7公里，新建改建城区管网659公里，累计完成基础设施和公共服务设施投资140亿元，滨河新区三水厂等9个水源工程和五县城生活污水处理厂建成投用，城区集中供热、供水供气、污水处理、垃圾收集实现全覆盖。加强历史文化名城保护，山西会馆、钟鼓楼、福音堂医院旧址等38项重点文物修缮工程全面完成。推广“四化”新农村建设模式，建成示范村146个、“美丽乡村”38个。大中型灌区节水改造、病险水库除险加固、河道治理、农村饮水安全等一批重点水利工程完成投资45亿元。整理高标准农田39万亩。新建改建国、省道及农村公路4468公里，公路通车总里程达到12488公里，建制村通畅率和班车通达率达91%和99%。兰新高铁建成通车，民航机场吞吐量累计达到20.7万人次。4G网络覆盖全部乡镇，光纤通村率达95%，信息基础设施建设水平跃居全国前列。

　　（四）着力强化生态建设，区域比较优势进一步彰显。坚持把强基础、保生态作为改善发展条件的战略选择，“一山一水”保护上升为国家战略，生态文明理念深入人心，全国主体功能区试点、生态文明工程试点市、水生态文明示范市、黑河湿地湖泊生态治理等重大国家战略和项目落地实施。累积争取国家投资16.1亿元，组织实施湿地恢复、天然林保护、退牧还草、三北防护林、防沙治沙、草原生态奖补等各类生态项目130项，完成人工造林36.5万亩、封育34万亩、义务植树2100多万株。荣获全国绿化模范城市和全省卫生城市，创建国家园林县城2个、省级园林县城4个。祁连山生态保护与建设综合治理规划、黑河湿地国家级自然保护区总体规划获批，黑河流域综合治理规划通过评审。黑河湿地湖泊纳入全国江河湖泊治理范围，张掖湿地列入国际重要湿地名录。张掖被确定为全国资源再生型试点城市。临泽、民乐被列为国家沙化土地封禁保护试点县。创建国家级、省级生态乡镇21个和53个。

五）着力强化民生保障，人民生活质量显著改善。坚持把惠民生、增福祉作为科学发展的根本取向，全面落实各项惠民政策，健全完善保障体系，社会各项事业协调发展。城乡居民人均可支配收入达到19673元和10823元，较“十一五”末分别增长81.23%和94.13%。财政用于民生的投入累计达到392亿元，占公共预算支出的79.8%。投入1.12亿元，办理为民承诺实事54件。城镇新增就业15.91万人。五大社会保险参保人数稳步增加，城镇居民基本医疗保险和城乡居民基本养老保险参保率分别达到98%和97%。新农合报销比例由“十一五”末的50.3%提高到61%。建成各类养老机构230个、保障性住房和棚户区改造房45607套，改造农村危旧房8.23万户，完成212万平方米既有居住建筑供热计量及节能改造任务。新建改建义务教育学校206所、幼儿园215所，临泽、高台、肃南通过国家县域义务教育基本均衡发展评估认定，九年义务教育巩固率达到99.48%，高考升学率稳居全省前列。张掖医专、市人民医院并入河西学院,部分医学专业实现“专升本”，省级财政、项目支持力度明显加大。新建医疗卫生业务用房1.15万平方米，城乡医疗卫生基础设施条件持续改善，重大疾病防控和基本公共卫生工作任务全面完成。人口自然增长率控制在5.5‰以内。强化食药安全监管，保障了人民群众饮食用药安全。实施各类科技项目311项，万人发明专利拥有量突破1件，培育高新技术企业16家。建成省级重点实验室、院士专家工作站12个，359项科技成果获得省级以上奖励，科技对经济增长的贡献率较“十一五”末提高5个百分点，市本级和六县区整体进入国家科技进步先进市县行列。积极创建国家公共文化服务体系示范区，完成投资22亿元。市级“三馆”、湿地博物馆、丹霞地质博物馆等重大文化项目及一大批综合文化站、村文化活动室、“乡村舞台”建成投用。全省体育与文化旅游融合发展示范区创建工作全面启动，中国户外运动名城建设列入国家体育总局规划。扎实推进“联村联户”、"1236"扶贫攻坚和精准扶贫脱贫工作，投入财政专项扶贫资金2.33亿元，减少贫困人口12.57万人。

　　（六）着力强化改革创新，发展环境明显优化。坚持把增活力、添动力作为改革创新的重要抓手，重点领域和关键环节改革有序推进。全面完成市县区政府和乡镇机构改革任务，事业单位分类改革稳步推进。在全省率先公布行政审批事项汇总清单，先后8批次取消、调整和下放行政审批事项452项，市县两级全面取消非行政许可审批。大力推进“三张清单一张网”建设，市直部门单位3216项审批权力、23525项责任事项、33477项追责情形公开发布。县级公立医院综合改革全面推行，基本药物制度实现全覆盖。积极开展农村综合配套改革，农村宅基地和集体建设用地使用权确权登记发证工作进展顺利，82.9万亩集体林地确权到户。全面公开财政预决算、部门预算、“三公”经费，财政资金管理使用更加规范高效。扎实推进公共资源交易市场化改革，交易额达292.2亿元。合理划分市、区城市规划建设、综合执法事权，管理体制进一步理顺。11亿“14张掖债”成功发行。引进甘肃银行、兰州银行和华安证券公司设立分支机构，95家企业在区域股权交易市场挂牌，新三板挂牌企业实现零的突破。严格执行中央八项规定精神、省委“双十条”和市委“八项要求”，“三公”经费逐年下降。调整腾退党政机关、事业单位办公用房6.1万平方米。深入开展党的群众路线教育实践活动和“三严三实”专题教育，健全各类规章制度769项，各级干部宗旨意识、纪律意识、规矩意识、法治意识明显增强。严格落实党风廉政建设主体责任，深化“庸懒散慢”专项治理，实行责任倒查机制和问责追究，政府执行力落实力不断提高。

2015年，面对持续加大的经济下行压力，我们紧紧抓住丝绸之路经济带建设重大战略机遇，更加突出转方式、调结构、稳增长、惠民生，力促三次产业协调发展，基本完成了市三届人大四次会议确定的目标任务。预计实现生产总值376.6亿元，增长7.5%。完成固定资产投资312.8亿元，增长13.5%。实现社会消费品零售总额148亿元，增长9%。大口径财政收入、公共财政预算收入分别完成53亿元和24.1亿元，增长10.2%和11.3% 。城镇居民人均可支配收入、农村居民人均可支配收入分别增长10%和11.5%。

　　（一）实施“双创示范”城市建设，增强了全社会创新活力。成功入围全国首批15个小微企业创业创新基地示范城市，争取国家支持资金6亿元。制定扶持政策措施，启动建设市级小微企业创富中心、电子商务创业园和张掖经济技术开发区创业大厦、民乐工业园区孵化园等一批创业创新基地，建成市级中小企业服务平台27家，6个基地被认定为“省级众创空间”，民乐生态工业园区创业基地被确定为第一批国家小微企业创业创新示范基地。与高等院校签订“双创示范”和“实训基地”合作协议，西部创客大学挂牌成立，“互联网小镇”筹建工作有序推进。在全省率先出台培育资本市场支持企业扩大直接融资办法，合作设立首期1亿元市级天使投资基金，扶持初创期、成长期小微企业加快发展。积极争取“互联网+”现代农业、高效物流、便捷交通等试点示范。开展“金张掖创新先锋”评选表彰活动，举办首届金张掖创业创新大赛及“赢在金张掖”电视公开赛，创业创新理念深入人心，支持创业、鼓励创新的鲜明导向已经形成。

　　（二）紧盯提质增效，巩固了农业比较优势。持续培育壮大优势主导产业和区域特色产业，完成农业投资33.7亿元，增长28.8%。种子企业联合上市工作全面启动，种业生产经营秩序整治工作受到农业部和省政府充分肯定。全国粮改饲和草牧业试点等一批重点项目获批实施，现代畜牧业全产业链试点工作扎实推进，奶肉牛、肉羊、生猪饲养量分别达到97万头、495万只和166万头，增长8%、7.3%和5.9%。农产品安全大市建设取得新的成效，甘州、临泽被命名为全省农产品安全县区。以龙头企业、专业合作社为引领的食用菌产业显示出良好的经济效益，辐射带动能力不断增强。奥林贝尔2000吨高效抗菌肽、嘉宏农牧3万吨蔬菜恒温气调库、金南瓜2000吨南瓜粉生产线等16个重点农业产业化项目建成投产。设立霍尔果斯商务代办处，签订农产品出口贸易合同12项7.5亿元，6家企业实现出口创汇118.2万美元，向西开放迈出坚实步伐。

　　（三）提升园区承载能力，实现了工业经济稳步回升。张掖国家级经济技术开发区循环化改造加快推进，民乐生态工业园区污水处理、标准化厂房、中药材孵化园等完成投资4.9亿元。市直和省属驻张单位帮扶园区“三个一”工作机制效果明显，争取各类资金5.67亿元，到位1.64亿元。新建续建工业项目207项，其中亿元以上46项，完成投资112亿元，增长12.8%。“两个园区”完成基础设施投资1.9亿元，引进企业15户，总数达到590户。龙源二期5万千瓦风力发电、华兴20万吨超细矿粉加工、汇能50兆瓦光伏发电、瑞和祥药业等70个重点项目建成投产。甘电投新能源5万千瓦风力发电、华西垃圾焚烧发电、晋昌源20万吨焦油加氢处理、8万吨棉浆粕等46个重点项目顺利推进。预计工业增加值增长5.5%，工业经济在市场环境不利的情况下回升向好，实现了平稳增长。

　　（四）深入实施“四大行动”，开创了旅游发展新局面。持续推进旅游景区晋等升级、全产业链打造、管理服务水平提升、全民旅游宣传“四大行动”，开工建设各类旅游项目73项，完成投资43.9亿元。张掖国家地质公园揭牌，张掖国家健身步道总体规划全面完成。新获批肃南裕固族民俗度假区、高台月牙湖景区2家国家4A级旅游景区，4A级景区达到15家，稳居全省之首。出台门票减免等优惠政策，冬季旅游市场活跃。发起成立河西五市旅游联盟，开通上海、杭州、合肥、宁波等地旅游包机和北京、成都等地旅游专列，冠名运行“金张掖号”、“裕固风情号”、“张掖丹霞号”、“甘州号”品牌列车。接待游客突破1500万人次，实现旅游综合收入76亿元，增长36%和36.2%。组织实施各类文化产业项目42项，完成投资22.2亿元。建成重点商流项目21项，实现投资18.8亿元。第二届“张交会”成功举办，盛况空前，影响力越来越大。电子商务异军突起，淘宝·特色中国张掖馆上线运营，电商企业和网店商家达到860多户，实现网上交易14.26亿元。快递业快速发展，年处理量突破600万件。

（五）加强交通水利电力建设，持续改善了基础设施条件。抢抓全省"6873"交通突破行动政策机遇，加快交通基础设施建设步伐，与中铁建集团签署245亿元战略投资协议，19条2095公里公路列入国网、省网规划。张扁高速、张肃高速、G312过境段等重大项目前期工作取得实质性成果，六东、甘平一级公路和黑河大桥开工建设，续建新建农村公路901公里，张掖客运中心站、高台汽车站主体工程完工。红山湾水库、农村饮水安全、河道治理、灌区节水改造等各类水利工程完成投资16.5亿元。总投资19.1亿元的黑河城区段治理一期工程开工建设。组织实施重点林业建设项目37项，完成投资3.57亿元。落实草原生态奖补资金2.19亿元。开工2座、改建2座110千伏变电站，农网改造升级完成建设任务，供电可靠性和保障能力进一步提升。

　　（六）加快基础设施改造提升，强化了城乡发展支撑。实施重点城市基础设施建设项目68项，完成投资10.3亿元。市区供水老旧管网改造、滨河新区天然气工程等49个项目开工建设，热电联产集中供热、污水处理二期等项目建成投用，制约市区扩容和滨河新区建设的瓶颈问题得到有效解决。高台国家新型城镇化综合试点，甘州党寨、山丹位奇省级新型城镇化试点稳步推进，沙井、南华等12个国家级重点镇和12个省列“千村美丽”示范村建设任务全面完成。获批国家智慧城市建设试点和“宽带中国”示范城市，加大骨干网络、光纤建设力度，通讯基础设施保障能力进一步增强。深入开展城乡环境综合整治和大气污染防治工作，空气、水环境质量指标控制在省政府下达的指标以内。扎实推进祁连山生态环境综合整治，影响生态环境的问题得到有效解决。

　　（七）全力推进精准扶贫，提高了民生保障水平。制定"1+19"本土化精准扶贫落地方案，把人财物和项目向3.49万扶贫对象和65个重点贫困村倾斜。整合资金6.21亿元，实现安全饮水、通村道路、危房改造、幼儿园、标准化卫生室五个全覆盖。发放精准扶贫专项贷款3.18亿元，当年整村脱贫目标顺利实现。省、市为民所办34件实事按期完成，各项惠民利民政策全面落实。甘州区全国养老示范基地、市第二人民医院开工建设，新建公租房4990套、棚户区改造房8649套，改造农村危旧房8009户，农村教职工和医务人员安居工程取得初步成效。

　　（八）致力建设法治张掖，保持了社会大局和谐稳定。严格落实重大行政决策事项法定程序，设立法律专家咨询委员会，依法决策、规范决策的机制进一步完善。强化执法监督，严格自由裁量程序，行政执法行为进一步规范。实施“平安细胞”工程，大力创建“平安示范村”，在全省率先建立平安指数预警发布制度，农村社区化、社区网格化管理深入推进，5个县区被省委、省政府命名为“平安县区”。健全完善矛盾纠纷多元排查调处、依法信访工作机制，集中开展信访积案化解“百日攻坚”行动，信访秩序持续好转。“六五”普法工作通过省级验收。扎实开展安全隐患排查整治“六打六治”专项行动，安全生产形势持续稳定向好。完善应急管理体系，加强防灾减灾能力建设，依法管理民族宗教事务，“平安张掖”建设成果进一步巩固，社会大局和谐稳定。

　　（九）持续深化改革，提升了政府效能。抓住抓准金融开放政策机遇，着力破解融资难题，银行信贷+企业直接融资+政府平台融资格局初步形成。8家金融机构推出40多种创新金融产品。与农发行、国开行签订战略合作协议，两行授信额度1100亿元，对接项目59项，分别审批项目贷款33.35亿元和1.24亿元。争取国家专项建设基金6.23亿元、政府债券资金16.7亿元。新组建3家政府投融资公司并实现实质性融资，城投、山水文旅投、农投、交投、水投五大投融资平台相互支撑、共同发展的格局基本形成。建立市级PPP项目库，34个项目纳入省级序列。改制成立张掖、临泽、高台、民乐农商银行。取消、调整、下放行政审批事项221项。全面推行机关事业单位工作人员养老保险制度改革，启动项目环评审批改革试点工作。土地承包经营权确权登记颁证工作深入推进，宅基地使用权抵押担保贷款取得实质性进展，水权水价改革试点工作进展顺利。“先照后证”、“三证合一”及全程电子化登记等商事制度改革措施全面落实，私营企业突破1万户。自觉接受人大法律监督、政协民主监督、新闻舆论监督和社会各界监督，办理答复人大代表建议97件、政协委员提案139件。严格落实党风廉政建设主体责任，加强重点领域、重点环节、重点部门监察审计，严肃查处违纪违规行为，政府效能进一步提升。

　　各位代表，过去的五年，是我们攻坚克难、砥砺奋进的五年，是经济发展速度最快、效益最好的五年，也是民生保障水平不断提高、群众得到实惠最多的五年。这些成绩的取得，是市委坚强领导、人大政协全力支持的结果，是全市各行各业、各族各界人民群众团结奋斗、开拓创新、苦干实干的结果。在此，我代表市人民政府，向关心、支持张掖发展的社会各界人士，向奋战在各条战线上的人大代表、政协委员、干部群众，向各民主党派、人民团体、中央和省属驻张单位，向离退休老同志、驻张部队、武警官兵、公安干警表示衷心的感谢并致以崇高的敬意！

　　回顾五年的奋斗历程，总结改革发展的经验教训，展望未来的发展前景，我们深切地感受到：必须不断深化市情认识，在实践探索中创新和完善发展思路，使张掖的发展更加符合时代潮流、更好地融入国家战略；必须始终紧抓第一要务不放松，在加快发展中破解难题，使张掖的发展步伐更快、质量效益更好；必须坚定不移地推进改革创新，激活全社会要素资源，使张掖的发展获得持久动力、增强发展后劲；必须牢固坚持为民服务宗旨，切实保障和改善民生，使张掖的发展惠及百姓、造福人民；必须牢固树立生态文明理念，坚持走生态经济发展之路，使张掖的发展更为科学、更可持续。

　　我们也清醒地认识到，全市经济社会发展中还存在诸多困难和不足。经济总量小，产业层次低，实现转型发展的难度和压力依然较大；大项目、好项目储备不足，新开工项目少，产业性重大项目接续不足，经济增长的内生动力依然不强；城镇居民人均可支配收入低于全国、全省平均水平；受市场和自然风险制约，农业持续增效、农民持续增收难度逐步加大；工业经济发展缓慢，吸纳就业能力较弱，骨干财源培植不足；市场发育度、开放度仍然较低，发展环境仍不宽松；各级干部的能力水平、纪律作风与创新发展的要求和人民群众的期望还有一定差距。这些问题，我们将采取针对性措施，切实加以解决。
